# Which System Variables Carry Robust Early Signs of Upcoming Phase Transition? An Ecological Example

**DOI:** 10.1371/journal.pone.0163003

**Published:** 2016-09-15

**Authors:** Ehsan Negahbani, D. Alistair Steyn-Ross, Moira L. Steyn-Ross, Luis A. Aguirre

**Affiliations:** 1 School of Engineering, The University of Waikato, Hamilton, Waikato, New Zealand; 2 Departamento de Engenharia Eletrônica, Universidade Federeal de Minas Gerais, Belo Horizonte, MG, Brazil; Consejo Nacional de Investigaciones Cientificas y Tecnicas, ARGENTINA

## Abstract

Growth of critical fluctuations prior to catastrophic state transition is generally regarded as a universal phenomenon, providing a valuable early warning signal in dynamical systems. Using an ecological fisheries model of three populations (juvenile prey *J*, adult prey *A* and predator *P*), a recent study has reported silent early warning signals obtained from *P* and *A* populations prior to saddle-node (SN) bifurcation, and thus concluded that early warning signals are not universal. By performing a full eigenvalue analysis of the same system we demonstrate that while *J* and *P* populations undergo SN bifurcation, *A* does not jump to a new state, so it is not expected to carry early warning signs. In contrast with the previous study, we capture a significant increase in the noise-induced fluctuations in the *P* population, but only on close approach to the bifurcation point; it is not clear why the *P* variance initially shows a decaying trend. Here we resolve this puzzle using observability measures from control theory. By computing the observability coefficient for the system from the recordings of each population considered one at a time, we are able to quantify their ability to describe changing internal dynamics. We demonstrate that precursor fluctuations are best observed using only the *J* variable, and also *P* variable if close to transition. Using observability analysis we are able to describe why a poorly observable variable (*P*) has poor forecasting capabilities although a full eigenvalue analysis shows that this variable undergoes a bifurcation. We conclude that observability analysis provides complementary information to identify the variables carrying early-warning signs about impending state transition.

## Introduction

Near a bifurcation point, a dynamical system may suddenly switch states without a significant change in the external drive forces. Such transitions are possible when the system has access to two (or more) stable states for the same control parameters. Bifurcation analysis describes altering fluctuation characteristics as a system moves towards a bifurcation point in response to a slowly varying control parameter, and how it switches state at or beyond the bifurcation point. Forecasting upcoming regime shifts is a vigorious research topic. Increased variance of noise-induced fluctuations is a well known universal early warning indicator of upcoming phase transitions, demonstrated in many model-based and experimentally obtained time series.

A 2013 article by Boerlijst et al [1] stated that catastrophic collapse in a structured three-variable predator-prey model can occur silently without occurrence of early warning signals in some model variables. They showed that warning signals only occur in the direction of the dominant eigenvector which is most strongly aligned with one of the variables. Since the other two variables were quiet prior to phase transition, they concluded that claims of the universality of early warning signals are not correct.

This paper elucidates the limitations of the method used in [1] to track the early warning signs. We demonstrate that it is required to perform a full eigenvalue analysis of the multidimensional model that undergoes regime shift. The analysis shows that not every system variable is expected to carry signs of critical slowing, yet this does not contradict the notion of the universality of critical fluctuations. We describe how the delayed rise of fluctuation variance of the second variable was overlooked, and will explain the requisite conditions for detection of critical fluctuations.

We extend the repertoire of analytic tools for detecting early warning signals by applying observability concepts adopted from control theory. This analysis allows us to identify which system variables are most representative of internal dynamics, and as a result carry information about bifurcation proximity.

The paper is structured as follows. We first recapitulate the fisheries model, determine its steady state behavior, and perform a linear stability analysis to extract the Jacobian matrix and corresponding eigenvalues, and identify possible bifurcations. We run a numerical simulation of the stochastic model and extract the fluctuation variances to confirm the results presented in [1], then cross-check against Ornstein-Uhlenbeck theoretical predictions. We compute the observability coefficients related to the three system variables and demonstrate how these coefficient describe the changes in fluctuation variance on approach to impending saddle-node induced regime shift.

## Methods and Results

### Model

The ecological model describes interaction dynamics between a predator and an age-structured prey composed of juvenile and adult developmental stages. The original model equations where the prey population is regulated through maturation and the predator feeds only on adult prey are as follows [2]:

$$\begin{array}{cc} \frac{dJ}{dt} & =f_{1}\left(J,A,P\right)=bA-\frac{\phi J}{1+dJ^{2}}-\mu_{J}J\\ \frac{dA}{dt} & =f_{2}\left(J,A,P\right)=\frac{\phi J}{1+dJ^{2}}-nAP-\mu_{A}A\\ \frac{dP}{dt} & =f_{3}\left(J,A,P\right)=cnAP-\mu_{P}P \end{array}$$ (1)

where *J*, *A*, and *P* are state variables describing the size of juvenile, adult and prey populations respectively. Here *bA* is the linear fecundity function with *b* as reproduction rate of adults. The nonlinear function *ϕJ*/(1 + *dJ*^2^) describes maturation of juvenile population with maturation rate of *ϕ*/(1 + *dJ*^2^) that goes to zero as juvenile population approaches infinity. *ϕ* is the maximum maturation rate of juvenile at low density, and *d* is the strength of exploitation competition among juvenile population. Parameter *n* is the attack rate of predator on adult and parameter *c* is the predator conversion efficiency. Death rates are *μ*_*J*_, *μ*_*A*_, *μ*_*P*_ for juvenile, adult and predator populations respectively.

As suggested in [2] a scaled form of this model is used here to reduce the number of parameters:

$$\begin{array}{cc} \frac{dJ}{dt} & =f_{1}\left(J,A,P\right)=bA-\frac{J}{1+J^{2}}-\mu_{J}J\\ \frac{dA}{dt} & =f_{2}\left(J,A,P\right)=\frac{J}{1+J^{2}}-AP-\mu_{A}A\\ \frac{dP}{dt} & =f_{3}\left(J,A,P\right)=cAP-\mu_{P}P \end{array}$$ (2)

where all rate parameters should now be interpreted as fractions of *ϕ*.

### Steady states of the model

The steady states of the model are determined by setting all time evolutions to zero and simultaneously solving the resulting steady state equations. Deriving the nontrivial solutions of steady state equations is not always possible in higher dimensional dynamical systems. New methods have been introduced recently to approximate fixed points of higher order stochastic systems based on corresponding deterministic mean-field approximation [3]. Due to mathematical simplicity of *P* equation in our model, we are able to directly solve the corresponding steady state equations. From the third equation in Eq (2) we have *P*(*Ac* − *μ*_*P*_) = 0 with *P* = 0 and *A* = *μ*_*P*_/*c* both satisfying the equation. Here we ignore the *P* = 0 trivial solution since this corresponds to complete elimination of predator population. Instead we select the nontrivial solution *A* = *μ*_*P*_/*c* which describes the equilibrium size of adult population. Using this equilibrium value in the second and third equations in Eq (2) we obtain:

$$\begin{array}{cc} 0 & =\frac{b\mu_{P}}{c}-\frac{J}{1+J^{2}}-\mu_{J}J\\ 0 & =\frac{J}{1+J^{2}}-P\frac{\mu_{P}}{c}-\frac{\mu_{A}\mu_{P}}{c} \end{array}$$ (3)

These equations can be rewritten as

$$c\mu_{J}J^{3}-b\mu_{P}J^{2}+\left(c\mu_{J}+c\right)J-b\mu_{P}=0$$ (4)

$$P=\frac{c}{\mu_{P}}\left[\frac{J}{1+J^{2}}-\frac{\mu_{A}\mu_{P}}{c}\right]$$ (5)

$$A=\frac{\mu_{P}}{c}$$ (6)

**Fig 1:**
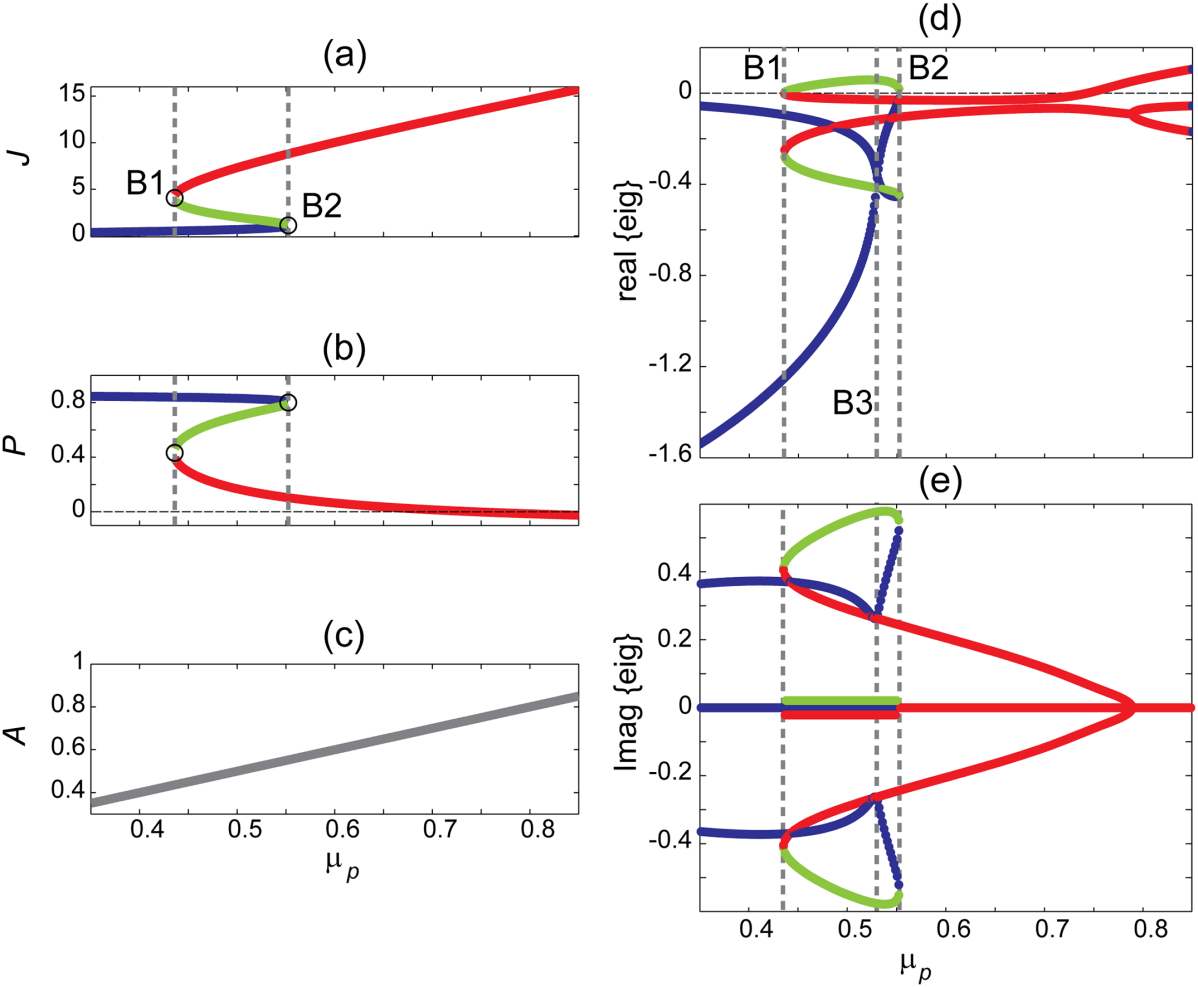
Steady-state diagram and corresponding eigenvalues of the fisheries model as a function of predator death rate *μ*_*P*_. (a-c) steady states showing a S-bend shape for *J* and *P* variables with color-coded lower (blue), middle (green) and upper (red) branches. Population *A* shows a linear trend with *μ*_*p*_. (d, e) Real and imaginary parts of linearized model. Saddle-node bifurcation points are marked B1 and B2 and indicated by vertical dashed grey lines. Model parameters are *c* = 1, *b* = 1, *μ*_*J*_ = 0.05, *μ*_*A*_ = 0.1. Eigenvalues are not relevant for $\mu_{P}\;≳\;0.74$ since predator population goes negative.

Eq (4) is a cubic polynomial in *J*, so its equilibrium values *J*° can be determined using the roots function in Matlab. Substitution in Eq (5) gives the corresponding value for *P*° and Eq (6) gives *A*°, the equilibrium values for predator and adult populations as a function of *μ*_*P*_ (death rate of predator population). Fig (1a)–(1c) shows the resulting steady state diagrams for each population. The juvenile and predator populations display multi-root regions while the adult density follows a linear trend over the full range of *μ*_*P*_ values. Two critical values of *μ*_*P*_ = *B*1, *B*2 on the border of multi-root region are indicated in Fig (1a) and (1b). Bifurcation points mark the locations where system dynamics undergoes a qualitative change. Eigenvalue analysis determines the stability properties and the types of bifurcation.

### Linear stability analysis

We perform a full eigenvalue analysis of linearized system following standard methods described in [4]. We linearize the model by writing:

$$\begin{array}{c} Z\left(t\right)=Z^{\text{o}}+\hat{Z}\left(t\right) \end{array}$$ (7)

where $\hat{Z}\left(t\right)$ is a small temporal perturbation, and *Z* ∈ {*J*, *A*, *P*}; *Z*° is the equilibrium point. It is informative to identify if $\hat{J},\hat{A}$, and $\hat{P}$ perturbations grow or decay to locate unstable and stable points. Replacing model variables with their perturbed forms, and using Taylor series expansions, Eq (2) can be written

$$\begin{array}{c} \frac{d\hat{J}}{dt}=f_{1}\left(\hat{J}+J^{\text{o}},\hat{A}+A^{\text{o}},\hat{P}+P^{\text{o}}\right)=f_{1}\left(J^{\text{o}},A^{\text{o}},P^{\text{o}}\right)+\frac{\partial f_{1}}{\partial J}{|}_{\text{o}}\hat{J}+\frac{\partial f_{1}}{\partial A}{{|}_{\text{o}}\hat{A}+\frac{\partial f_{1}}{\partial P}|}_{\text{o}}\hat{P}+\cdots \\ \frac{d\hat{A}}{dt}=f_{2}\left(\hat{J}+J^{\text{o}},\hat{A}+A^{\text{o}},\hat{P}+P^{\text{o}}\right)=f_{2}\left(J^{\text{o}},A^{\text{o}},P^{\text{o}}\right)+\frac{\partial f_{2}}{\partial J}{|}_{\text{o}}\hat{J}+\frac{\partial f_{2}}{\partial A}{{|}_{\text{o}}\hat{A}+\frac{\partial f_{2}}{\partial P}|}_{o}\hat{P}+\cdots \\ \frac{d\hat{P}}{dt}=f_{3}\left(\hat{J}+J^{\text{o}},\hat{A}+A^{\text{o}},\hat{P}+P^{\text{o}}\right)=f_{3}\left(J^{\text{o}},A^{\text{o}},P^{\text{o}}\right)+\frac{\partial f_{3}}{\partial J}{|}_{\text{o}}\hat{J}+\frac{\partial f_{3}}{\partial A}{{|}_{\text{o}}\hat{A}+\frac{\partial f_{3}}{\partial P}|}_{\text{o}}\hat{P}+\cdots \end{array}$$ (8)

where the higher order terms are neglected since $\hat{Z}$ is small. Noting that *f*_1,2,3_(*J*°, *A*°, *P*°) = 0, we obtain,

$$\begin{array}{c} \frac{d\hat{J}}{dt}=\frac{\partial f_{1}}{\partial J}{|}_{\text{o}}\hat{J}+\frac{\partial f_{1}}{\partial A}{{|}_{\text{o}}\hat{A}+\frac{\partial f_{1}}{\partial P}|}_{\text{o}}\hat{P}\\ \frac{d\hat{A}}{dt}=\frac{\partial f_{2}}{\partial J}{|}_{\text{o}}\hat{J}+\frac{\partial f_{2}}{\partial A}{{|}_{\text{o}}\hat{A}+\frac{\partial f_{2}}{\partial P}|}_{\text{o}}\hat{P}\\ \frac{d\hat{P}}{dt}=\frac{\partial f_{3}}{\partial J}{|}_{\text{o}}\hat{J}+\frac{\partial f_{3}}{\partial A}{{|}_{\text{o}}\hat{A}+\frac{\partial f_{3}}{\partial P}|}_{\text{o}}\hat{P} \end{array}$$ (9)

to describe the time evolution of the perturbations $\hat{J},\hat{A},\hat{P}$. Expressing in matrix form,

$$\begin{array}{c} \frac{d}{dt}\left[\begin{array}{c} J(t)\\ A(t)\\ P(t) \end{array}\right]=\tilde{J}\left[\begin{array}{c} J(t)\\ A(t)\\ P(t) \end{array}\right] \end{array}$$ (10)

where

$$\begin{array}{c} \begin{array}{c} \tilde{J}=\left[\begin{array}{ccc} \frac{\partial f_{1}}{\partial J} & \frac{\partial f_{1}}{\partial A} & \frac{\partial f_{1}}{\partial P}\\ \frac{\partial f_{2}}{\partial J} & \frac{\partial f_{2}}{\partial A} & \frac{\partial f_{2}}{\partial P}\\ \frac{\partial f_{3}}{\partial J} & \frac{\partial f_{3}}{\partial A} & \frac{\partial f_{3}}{\partial P} \end{array}\right]=\left[\begin{array}{ccc} -\frac{1-J^{2}}{{\left(1+J^{2}\right)}^{2}}-\mu_{J} & b & 0\\ \frac{1-J^{2}}{{\left(1+J^{2}\right)}^{2}} & -(P+\mu_{A}) & -A\\ 0 & cP & cA-\mu_{P} \end{array}\right] \end{array} \end{array}$$ (11)

is the *Jacobian matrix* evaluated at the equilibrium point (*J*°, *A*°, *P*°) and *f*_*i*_, *i* ∈ {1, 2, 3} are system functions defined in Eq 2. The exponential time-course for small perturbations away from steady state can be predicted from the eigenvalues of $\tilde{J}$ [5]. The equilibrium is stable when all eigenvalues have negative real parts, otherwise the equilibrium is unstable. Close to equilibrium the system dynamics is determined by the dominant eigenvalue, i.e., the eigenvalue whose real part is least negative. See Fig 1(d).

The lower branch of *J* steady-state diagram (top branch of *P*) is a stable focus-node since the system has one real negative eigenvalue and a pair of complex conjugate eigenvalues with negative real part. Note that the focus is dominant for *μ*_*P*_ ≤ *B*_3_ but the node takes over for *μ*_*P*_ ≥ *B*_3_. The top branch of *J* (bottom branch of *P*) forms stable focus-nodes for $\mu_{P}≲0.74$ where the node aspect is dominant. In contrast, all points on the midbranch are unstable saddle-focus equilibria forming a separatrix between upper and lower branches.

We identify three critical *μ*_*P*_ values:

1. *μ*_*B*_1__ = 0.43525: a stable focus-node collides with an unstable saddle-focus. This is simply a saddle-node bifurcation at the left-hand turning point.
2. *μ*_*B*_2__ = 0.55281: saddle-node bifurcation at right-hand turning point.
3. *μ*_*B*_3__ = 0.53017: dominance is swapped between focus and node components. Stability is not modified, so a bifurcation does not happen here.

Having identified bifurcation points (*B*_1_ and *B*_2_) and special point (*B*_3_), we run a series of stochastic numerical simulations, then validate against theoretical predictions.

### Noise-induced fluctuations prior to bifurcation

**Fig 2:**
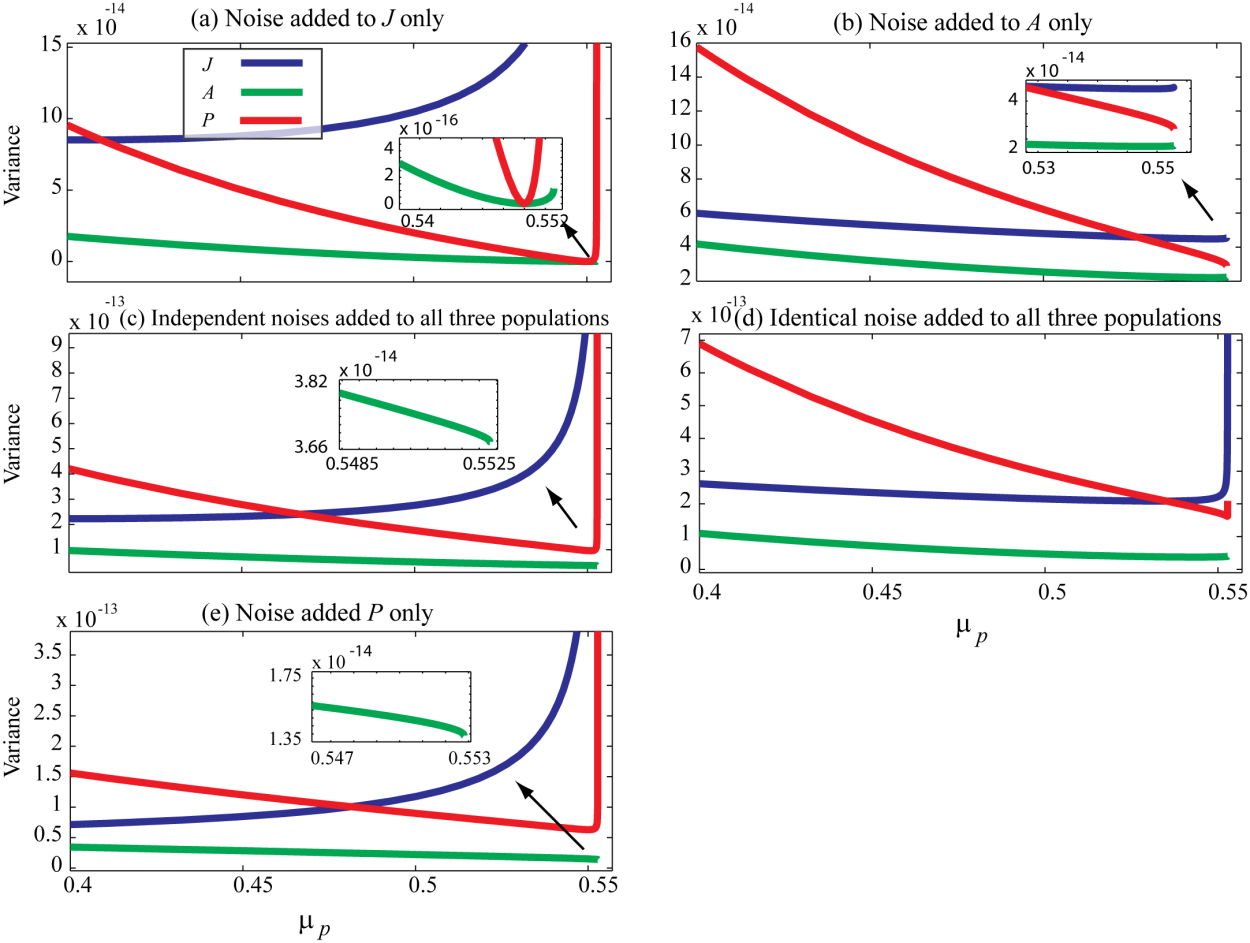
Numerically obtained fluctuation variance of model variables prior to saddle-node bifurcation. (a–d) For each value of *μ*_*P*_, starting with *μ*_*P*_ = 0.4, the model is simulated for 60,000 time units with resolution of 0.01 time units, from which the population variances are computed. *μ*_*P*_ is incremented geometrically towards the catastrophic collapse at *μ*_*P*_ = *μ*_*B*_2__. Death rates are perturbed every time unit using white noise with standard deviation of *σ*_noise_ = 2 × 10^−6^. (a) Noise added to the juvenile population (b) Noise added to the adult population (c). Independent noise added to all three populations. (d) Identical, fully correlated, noise added to all three populations. (e) An extra experiment where noise is added to *P* only population.

We repeat the numerical experiments described in [1] but, rather than displaying coefficient of variation (standard deviation divided by mean), in Fig 2 we plot the unscaled fluctuation variance as a function of *μ*_*p*_. The coefficient of variation is not ideal for tracking fluctuations since changes in the mean can confound changes in fluctuation amplitude; the latter is where the dynamic information resides. We first analyze the case when the noise is added to *J* population only as shown in Fig 2(a). From eigenvalue analysis we estimate the mortality rate at the right-hand saddle-node point as *μ*_*P*_ = *μ*_*B*_2__ ≈ 0.55280625013933332. We use geometrically spaced *μ*_*P*_ values to closely approach this catastrophic collapse point while noise is added to the death rate of *J* population in the same way as described by Boerlijst et al:

$$\begin{array}{cc} \frac{dJ}{dt} & =bA-\frac{J}{1+J^{2}}-\left(\mu_{J}+a_{1}\xi_{1}\left(t\right)\right)J \end{array}$$ (12)

where *a*_1_ is a coefficient to ensure that the noise amplitude is small, and *ξ*_1_(*t*) represents a zero-mean, Gaussian-distributed white-noise process. We first calculate the steady state, and then use this as the initial value in an Euler-based numerical update of the differential equations using time steps of Δ*t* = 0.01. The variance is extracted for each *μ*_*P*_ value and trends are plotted for 0.4 ≤ *μ*_*p*_ ≤ *μ*_*B*_2__.

Results are shown in Fig 2(a). Despite the general similarity with those reported in [1], there are subtle but important differences. We see that the fluctuation variance increases prior to catastrophe in *all three* populations, Fig 2(a). This increase is significant for juvenile and predator populations and very weak for adult population. We found that capturing the growth of fluctuation variance for adult and predator populations is numerically challenging, and requires:

1. Precise identification of control parameter value (*μ*_*P*_) at phase transition.
2. Performing numerical experiments in close vicinity to catastrophe. We chose geometrically spaced *μ*_*p*_ values to closely approach the *B*_2_ saddle-node,
  $$\mu_{p}=\mu_{B_{2}}\left(1+\epsilon \right),\;\epsilon =1/4^{j}\;\text{w}ith\;j=1,2,\ldots \;25$$ (13)
3. Application of considerably smaller amplitude white noise when performing the experiments close to phase transition. We used very small amplitude noise with standard deviation *σ* = 6.317 × 10^−7^ compared to *σ* = 0.005 used in [1], otherwise the noise-induced fluctuations are strong enough to cause a jump transition in the system, preventing us from dwelling close to bifurcation.

Fig 2 shows that fluctuation variance grows significantly prior to SN point for *J* and *P* population, and has a tiny or zero growth for *A* population regardless of the way noise is added to the system. The exception is when noise is added to the A population only (Fig 2(b)): for this case, none of the populations exhibit fluctuation growth. The results of an extra experiment where the noise is added to *P* only population is displayed in Fig 2(e).

To verify the accuracy of our simulations, we transform the stochastic model equations into Ornstein-Uhlenbeck (OU) form. Then we compare the theoretically-predicted OU statistics against the corresponding values extracted from numerical simulations.

### Ornstein-Uhlenbeck (OU) analysis

We include additive white noise in all three equations to transform model (2) to a stochastic form suitable for OU analysis,

$$\begin{array}{cc} \frac{dJ}{dT} & =bA-\frac{J}{1+J^{2}}-\mu_{J}J+a_{1}\xi_{1}\left(t\right)\\ \frac{dA}{dt} & =\frac{J}{1+J^{2}}-AP-\mu_{A}A+a_{2}\xi_{2}\left(t\right)\\ \frac{dP}{dt} & =APc-\mu_{P}P+a_{3}\xi_{3}\left(t\right) \end{array}$$ (14)

where *a*_1,2,3_ are scaling constants that ensure that the fluctuations are small, and *ξ*_1,2,3_ are independent zero-mean, Gaussian-distributed white-noise sources [6],

$$\begin{array}{c} \left\langle \xi \left(t\right)\right\rangle =0\;,\;\left\langle \xi_{m}\left(t\right)\xi_{n}\left(t^{\prime }\right)\right\rangle =\delta_{mn}\delta \left(t-t^{\prime }\right) \end{array}$$ (15)

where *δ*_*mn*_ is the dimensionless Kronecker delta, *δ*(⋅) is the dirac delta with a dimensionless total area under its curve equal to one, and the 〈⋅⋅⋅〉 represents the ensemble average over time. Noise samples (implemented by Matlab’s randn function) are scaled as

$$\begin{array}{c} \xi \left(t\right)=\frac{R_{n}\left(0,1\right)}{\sqrt{\Delta t}} \end{array}$$ (16)

where $R_{n}\left(0,1\right)$ describes a zero-mean, unit-variance Gaussian random number generator; the scaling by $\sqrt{\Delta t}$ ensures that *ξ*(*t*) tends to an infinite-variance white noise in the limit Δ*t* → 0 [6, 7, 8]. (Notice that the noise is not added to the mortality rates of populations as implemented by in Boerlijst et al [1] and shown in Eq (12)). We arrange the model equations in matrix form:

$$\begin{array}{c} \frac{d}{dt}\left[\begin{array}{c} J\\ A\\ P \end{array}\right]=\tilde{J}\left[\begin{array}{c} J\\ A\\ P \end{array}\right]+\left[\begin{array}{c} a_{1}\xi_{J}\\ a_{2}\xi_{A}\\ a_{3}\xi_{P} \end{array}\right] \end{array}$$ (17)

with $\tilde{J}$ as the Jacobian matrix defined in Eq (11). We recast into standard OU form, [9, 10]:

$$\frac{d}{dt}\left[\begin{array}{c} J\\ A\\ P \end{array}\right]=-\tilde{A}\left[\begin{array}{c} J\\ A\\ P \end{array}\right]+\sqrt{D}\left[\begin{array}{c} \xi_{J}\\ \xi_{A}\\ \xi_{P} \end{array}\right]$$ (18)

where $\tilde{A}=-\tilde{J}$ is the *drift matrix* and **D** is a diagonal 3 × 3 *diffusion matrix*

$$\begin{array}{c} D=\left[\begin{array}{ccc} a_{1}^{2} & 0 & 0\\ 0 & a_{2}^{2} & 0\\ 0 & 0 & a_{3}^{2} \end{array}\right] \end{array}$$ (19)

Based on well documented OU statistics [9, 10], we can immediately write down theoretical expressions for covariance matrix of the model. The stationary *covariance matrix* **Σ** of an OU process is defined by

$$A\Sigma +\Sigma A^{\text{T}}=D$$ (20)

where **Σ** is the 3 × 3 stationary covariance matrix

$$\begin{array}{c} \Sigma =\left[\begin{array}{ccc} \text{var}(J) & \text{cov}(J,A) & \text{cov}(J,P)\\ \text{cov}(A,J) & \text{var}(A) & \text{cov}(A,P)\\ \text{var}(P,J) & \text{cov}(P,A) & \text{var}(P) \end{array}\right] \end{array}$$ (21)

from which one can extract the theoretical variance predictions. Noting that Eq (20) is the continuous Lyapunov equation, one can derive **Σ** as:

$$\begin{array}{c} \text{vec}\left(\Sigma \right)={\left(I⊗A+A⊗I\right)}^{-1}\;\text{vec}\left(D\right) \end{array}$$ (22)

where vec() represents the vectorised form of a matrix and ⊗ is the Kronecker product [11]. The vectorization operation is defined as the stacking of the columns of a matrix into a vector. This method is a generalization of the approach suggested by Gardiner which is limited to 2 × 2 matrices [10].

**Fig 3:**
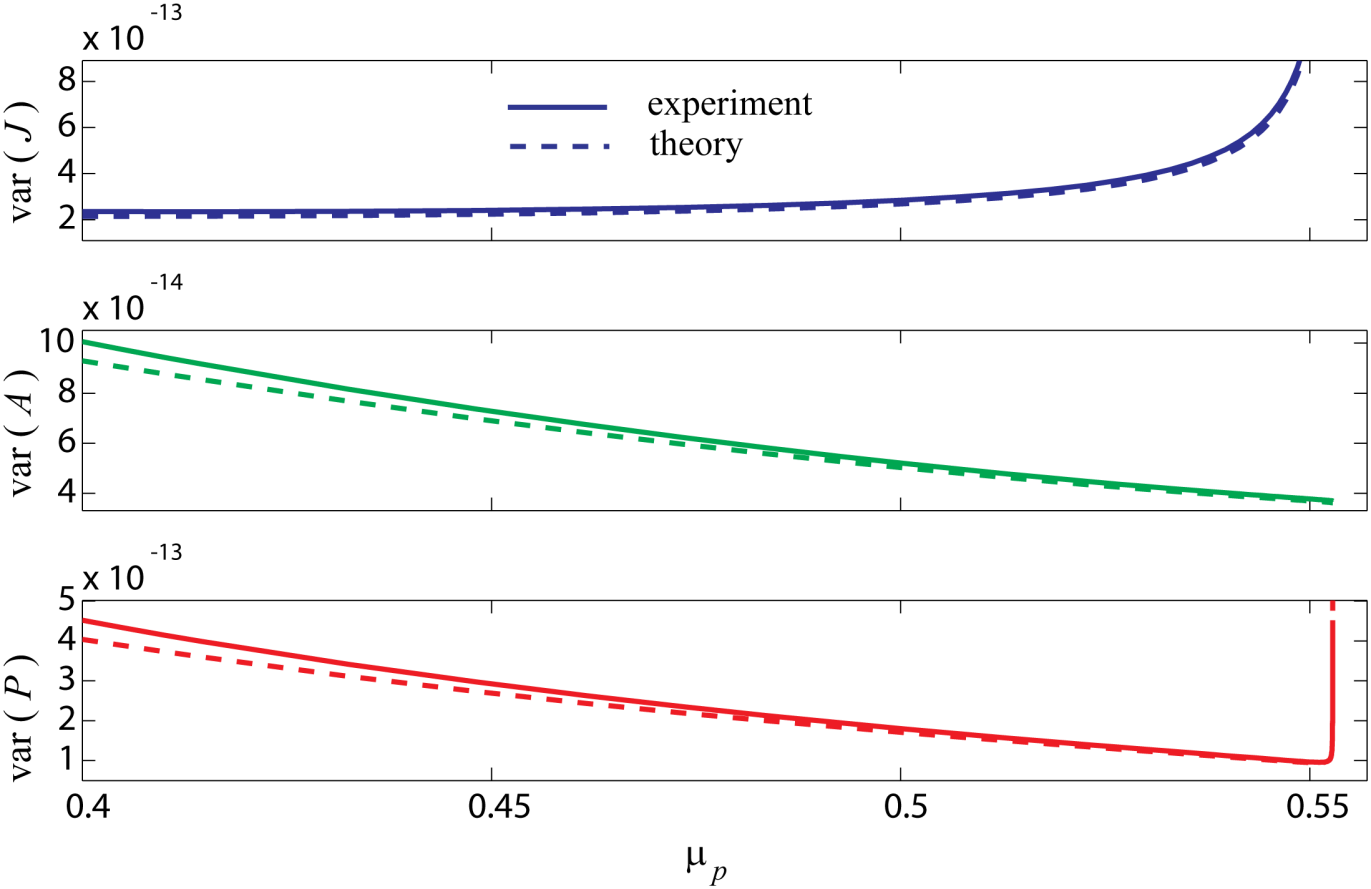
Fluctuation variance prior to saddle-node bifurcation with independent noises added to all three populations. Experimental and theoretical variances are plotted for all three populations while approaching catastrophe. A fixed step Euler method with Δ*t* = 0.01 is used for numerical simulations each for 6000 time units. Starting with *μ*_*P*_ = 0.4, it is incremented towards the saddle-node point at *μ*_*B*_2__. The distance from bifurcation point is geometrically reduced enabling more experiments close to bifurcation. Three independent white noise sources are added to each population as described in Eqs (14)–(16) with standard deviation of *σ*_noise_ = 2 × 10^−6^.

We use Eq (22) to compute the theoretical covariance matrix. Eq (21) shows that the diagonal elements of this matrix represent the individual fluctuation variances of each of the three populations. We perform similar experiments as shown in Fig 2(c) where independent noises are added to all three populations and plot the numerically obtained variances as solid lines in Fig 3(a)–3(c); theoretical predictions are superimposed as dashed lines. Good agreement between theory and experiment is evident in all three populations. Variance of *J* fluctuations increases towards the SN point as shown in Fig 3(a). Variances of *A* and *P* populations show a decreasing behaviour, but *P* variance recovers and increases significantly close to catastrophe as shown in (b), (c).

One can perform similar analysis when the noise is added to only *J* or *P* population. The qualitative form of the results is not strongly sensitive to the number of independent noise sources, e.g., compare the three-noise case of Fig 3 with S1 and S2 Figs.

These results show that although a saddle-node bifurcation occurs in this three-dimensional dynamical system, not all system variables display early warning indicators. Considering the steady-state diagrams of Fig 1, one notices that *J* and *P* populations switch to a new state at bifurcation points while *A* variable does not. The linear steady state diagram for *A* population does not support bifurcation, so it is not expected to show critical fluctuations.

On the other hand one might expect to capture reliable early warning signs on both *J* and *P* fluctuations due to their similar multi-root steady-state diagrams, but the results do not support this expectation. While the *J* population demonstrates consistent growth towards the SN point, the *P* population does not. Instead, we only observe significant fluctuation growth very close to SN.

**Fig 4:**
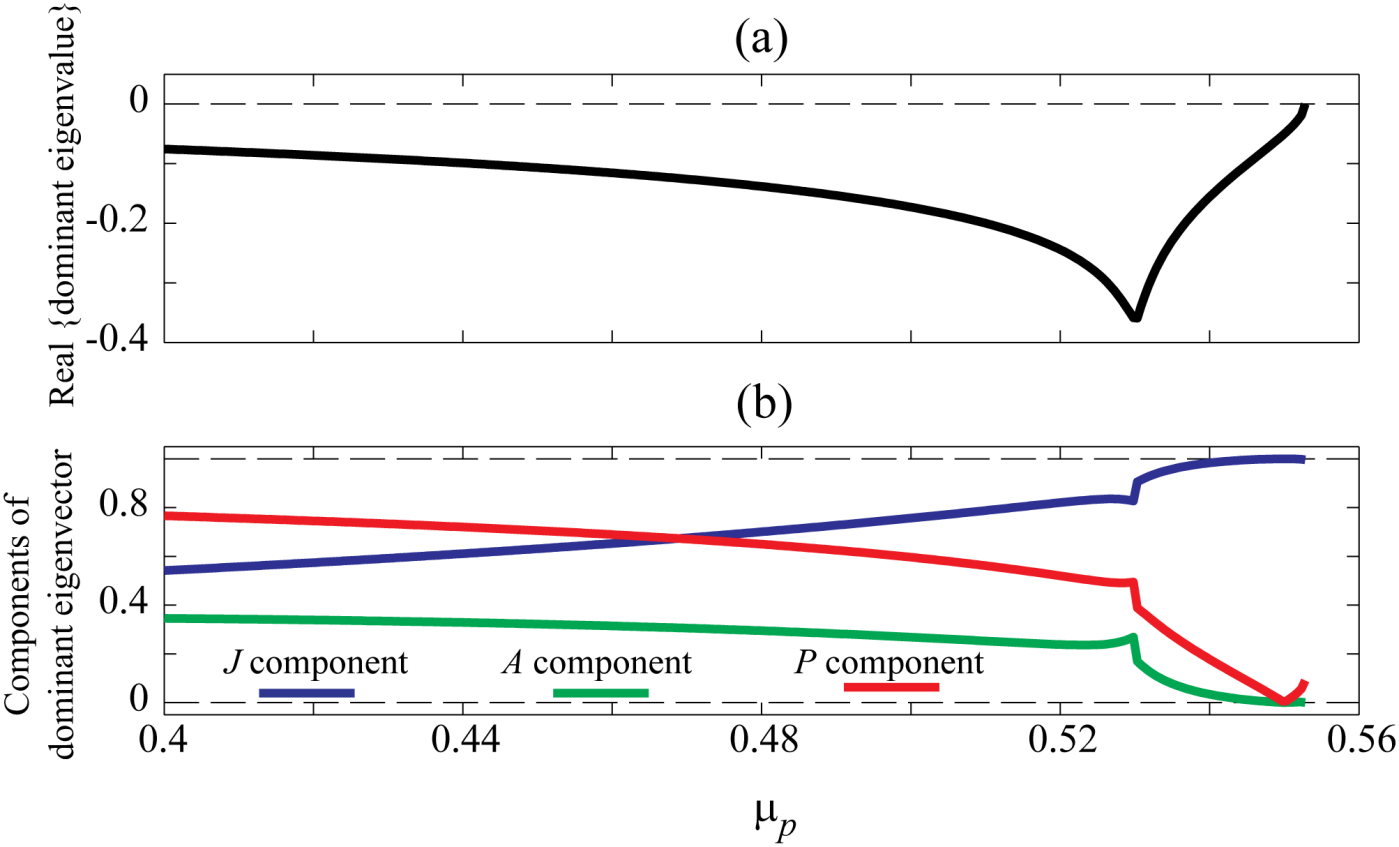
Tracking of dominant eigenvalue and its decomposed eigenvector towards saddle-node bifurcation point. (a) The real part of dominant eigenvalue and (b) the corresponding decomposed eigenvector as a function of predator mortality rate *μ*_*P*_.

Boerjlist et al. [1] used the concept of dominant eigenvector to explain the strong growth in *J* fluctuations and apparent invisibility of *P* fluctuations. We tested this idea by extracting the dominant eigenvalue and corresponding eigenvector components for a broad range of *μ*_*P*_ values while approaching the *B*_2_ saddle-node point. Fig 4 displays the dominant eigenvalue (previously displayed as part of Fig 1(d)) and the corresponding eigenvector decomposed into its *J*, *P* and *A* components. Close to state transition, the dominant eigenvector has a significant *J* component, a small *P* component, and an almost zero *A* component.

Although behaviour of the eigenvalue components matches the variance trends—and Boerlijst et al [1] reported similar findings—one may still ask the important question: Why do the *P* fluctuations not increase all the way towards SN point? Since SN bifurcation can occur in one-dimensional systems [12], perhaps it is the case that *J* is the only variable in control of SN bifurcation in this model. We will demonstrate that observability analysis can provide insights for this argument.

### Observability analysis

Inferring whole system dynamics based on measuring a single time series of the system is a challenging problem. *Observability* analysis reveals which variable (or combination of variables) best represents internal dynamics. See S1 Appendix for a general description of observability analysis. Here we compute the *observability coefficients* for the system when monitored through *J*, *P*, and *A* populations to quantify the system observability from individual variables. The observability coefficient is derived from the observability matrix ${\tilde{O}}_{x}\;\left(x\in \left\{J,P,A\right\}\right)$ constructed from either Lie derivatives or a coordinate transformation that maps from the system to a nominated single variable [13]. Both procedures are mathematically the same, resulting in the same observability matrix. Here we construct the observability matrices from Lie derivatives. The same observability matrices can be obtained using coordinate transformation as shown in S2 Appendix. The Lie derivatives of the system are

$$\begin{array}{c} \begin{array}{cc} L_{f_{1}} & =\frac{\partial f_{1}}{\partial J}f_{1}+\frac{\partial f_{1}}{\partial A}f_{2}+\frac{\partial f_{1}}{\partial P}f_{3}\\ & =\left[-\frac{1-J^{2}}{{\left(1+J^{2}\right)}^{2}}-\mu_{J}\right]\left[bA-\frac{J}{1+J^{2}}-\mu_{J}J\right]+b\left[\frac{J}{1+J^{2}}-AP-\mu_{A}A\right]\\ L_{f_{2}} & =\frac{\partial f_{2}}{\partial J}f_{1}+\frac{\partial f_{2}}{\partial A}f_{2}+\frac{\partial f_{2}}{\partial P}f_{3}\\ & =\frac{1-J^{2}}{{\left(1+J^{2}\right)}^{2}}\left[bA-\frac{J}{1+J^{2}}-\mu_{J}J\right]-\left(P+\mu_{A}\right)\left[\frac{J}{1+J^{2}}-AP-\mu_{A}A\right]\\ & \;\;\;\;-A(cAP-\mu_{P}P)\\ L_{f_{3}} & =\frac{\partial f_{3}}{\partial J}f_{1}+\frac{\partial f_{3}}{\partial A}f_{2}+\frac{\partial f_{3}}{\partial P}f_{3}\\ & =cP\left(\frac{J}{1+J^{2}}-AP-\mu_{A}A\right)+P{\left(cA-\mu_{P}\right)}^{2} \end{array} \end{array}$$

with *f*_*i*_, *i* ∈ {1, 2, 3} previously defined in Eq 2. The next step is to construct matrix ${\tilde{J}}^{2}$ by taking derivatives of $L_{f_{1}},L_{f_{2}}$ and $L_{f_{3}}$ with respect to *J*, *A* and *P* variables

$$\begin{array}{c} {\tilde{J}}^{2}=\left[\begin{array}{ccc} \frac{\partial L_{f_{1}}}{\partial J} & \frac{\partial L_{f_{1}}}{\partial A} & \frac{\partial L_{f_{1}}}{\partial P}\\ \frac{\partial L_{f_{2}}}{\partial J} & \frac{\partial L_{f_{2}}}{\partial A} & \frac{\partial L_{f_{2}}}{\partial P}\\ \frac{\partial L_{f_{3}}}{\partial J} & \frac{\partial L_{f_{3}}}{\partial A} & \frac{\partial L_{f_{3}}}{\partial P} \end{array}\right] \end{array}$$

We are interested in observability analysis via each individual system variable, so define *measurement* vectors,

$$\begin{array}{c} \begin{array}{ccc} C_{1}=\left[1\;0\;0\right], & C_{2}=\left[0\;1\;0\right], & C_{3}=\left[0\;0\;1\right] \end{array} \end{array}$$

corresponding to *J*, *A* and *P* variables respectively. Then the state-dependent observability matrix of each variable is

$$\begin{array}{c} \begin{array}{ccc} O_{J}=\left[\begin{array}{c} C_{1}\\ C_{1}\tilde{J}\\ C_{1}{\tilde{J}}^{2} \end{array}\right], & O_{A}=\left[\begin{array}{c} C_{2}\\ C_{2}\tilde{J}\\ C_{2}{\tilde{J}}^{2} \end{array}\right], & O_{P}=\left[\begin{array}{c} C_{3}\\ C_{3}\tilde{J}\\ C_{3}{\tilde{J}}^{2} \end{array}\right]. \end{array} \end{array}$$ (23)

These matrices can be constructed for every point at space space, but we choose to compute them only at stable steady states in order to confine our study to ecologically significant stable states. A system is defined to be observable via variable *x* if the observability matrix $O_{x}$ is full rank, otherwise not observable [14]. Equivalently if $O_{x}^{T}O_{x},\;x\in \left\{J,\;A,\;P\right\}$ is nonsingular (does not have a zero eigenvalue), then the system is observable, otherwise not. In practice, an observable system may gradually lose observability due to a varying system parameter. Aguirre et al. [13] developed a method to quantify degree of observability using *observability coefficient*. Following their method to compute the observability coefficient through individual system variables one should first extract the state dependent observability measure as

$$\begin{array}{c} \delta_{x}\left(J(t),A(t),P(t)\right)=\frac{|\lambda_{\text{min}}\left[O_{x}^{T}O_{x}\right]|}{|\lambda_{\text{max}}\left[O_{x}^{T}O_{x}\right]|},\;\;\;x\in \left\{J,\;A,\;P\right\} \end{array}$$ (24)

where $\lambda_{\text{max}}\left[O_{x}^{T}O_{x}\right]$ is the maximum eigenvalue of matrix $O_{x}^{T}O_{x}$ estimated at point (*J*(*t*), *A*(*t*), *P*(*t*)) (likewise for λ_min_). According to this definition 0 ≤ *δ*_*x*_ ≤ 1 and the lower bound is reached when the system is unobservable for variable *x*. The next step is to compute the observability coefficient ${\bar{\delta }}_{x}$ (which is a constant) as the time average of *δ*_*x*_(*J*(*t*), *A*(*t*), *P*(*t*)) along the trajectory (*J*(*t*), *A*(*t*), *P*(*t*)), *t* ∈ {*t*_initial_, *t*_final_}. Confining our analysis to stable steady states, we extract ${\bar{\delta }}_{x}$ locally without a need for time averaging, giving a local measure of stability:

$$\begin{array}{c} {\bar{\delta }}_{x}\left(J^{\text{o}},A^{\text{o}},P^{\text{o}}\right)=\frac{|\lambda_{\text{min}}\left[O_{x}^{T}O_{x}\right]|}{|\lambda_{\text{max}}\left[O_{x}^{T}O_{x}\right]|},\;\;\;x\in \left\{J,\;A,\;P\right\} \end{array}$$ (25)

where observability matrices are computed at steady states. We use this coefficient to determine the observability of the fisheries model while gradually approaching the *B*_2_ saddle-node point by increasing the mortality rate of the prey population.

**Fig 5:**
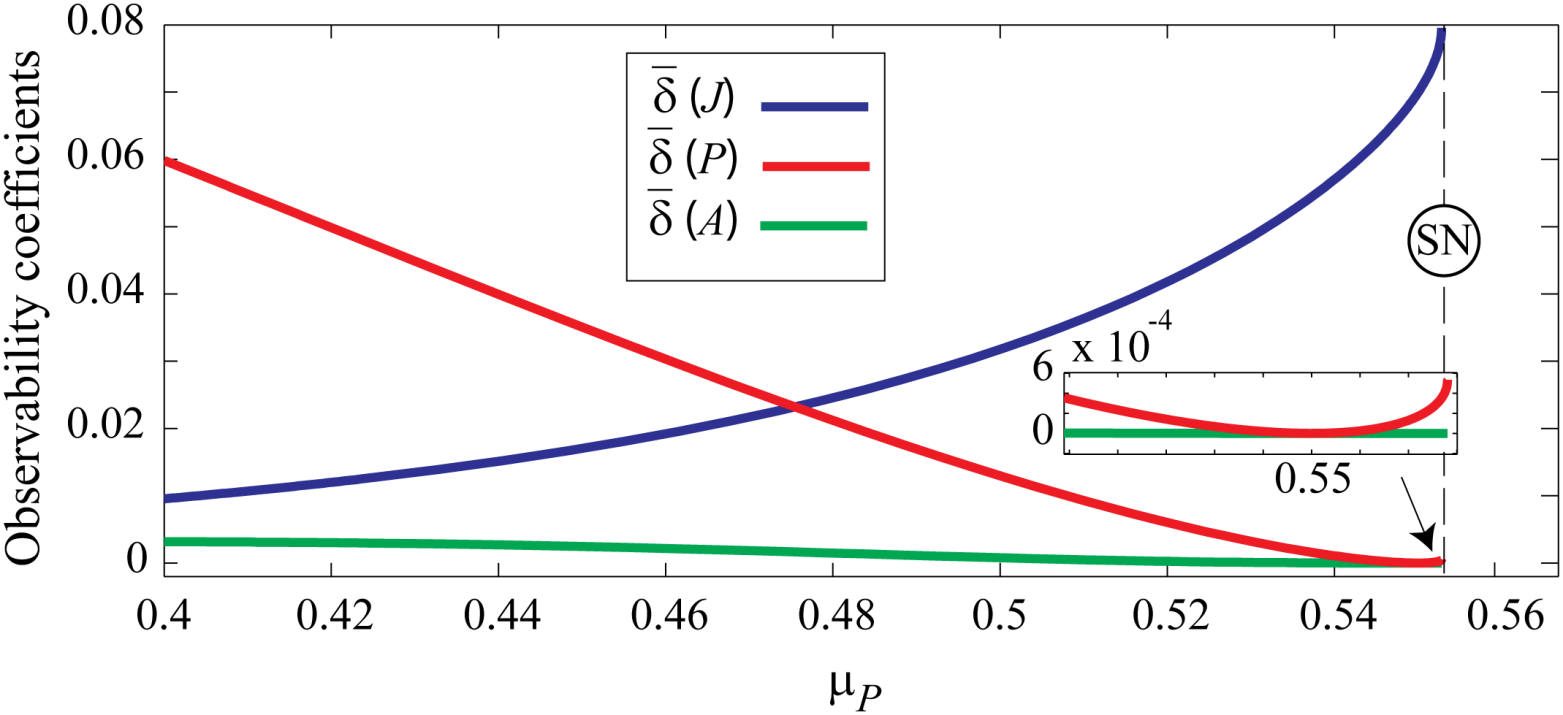
Observability coefficients of fisheries model. Observability coefficient of system variables $\bar{\delta }\left(J\right),\bar{\delta }\left(P\right)$, and $\bar{\delta }\left(A\right)$ as a function of *μ*_*P*_ calculated using the definition in Eq (24). Lie derivatives are used to construct the observability matrix from which the observability coefficients are calculated. See text for details. A geometrically spaced vector of predator mortality rate is used to cover the range 0.4 ≤ *μ*_*P*_ ≤ *μ*_*B*_2__.

We extract the observability coefficients using observability matrices obtained by Lie derivatives. The results are shown in Fig 5. ${\bar{\delta }}_{J}$ and ${\bar{\delta }}_{P}$ cross at *μ*_*P*_ ≈ 0.475 showing that for lower values of the predator mortality rate it is best to measure *P* whereas for higher rates of *μ*_*P*_ it is best to measure *J* if one is to monitor the internal dynamics. Focusing at the close vicinity of SN point, while the system is significantly observable from *J* population ($\bar{\delta }\left(J\right)\approx 0.08$), the observability coefficients of *P* and *A* populations are $\bar{\delta }\left(P\right)\approx 6\times 10^{-4}$ and $\bar{\delta }\left(A\right)\approx 0$. These values indicate that the system is mainly observable from *J* prior to SN point. The coefficient trends over the *μ*_*P*_ range show a decrease in system observability via $\bar{\delta }\left(P\right)$ and $\bar{\delta }\left(A\right)$ but an increase for $\bar{\delta }\left(J\right)$. Notice that $\bar{\delta }\left(P\right)$ recovers slightly just prior to SN point as shown in the inset. These trends are in agreement with variance of fluctuations as displayed in Figs (2a), (2c) and (2e) and 3. The small observability coefficients of *P* and *A* variables explain the challenging task of capturing signs of slowing down on these variables in numerical simulations. This implies that signs of slowing down would be very hard to observe on predator or adult populations in field or even in controlled laboratory setups.

## Discussion

Boerjlist et al. have shown that not every variable of a structured prey-predator ecological model presents precursors of upcoming saddle-node collapse [1]. They performed a series of numerical simulations in which different noise types were added to model variables in a search for early signs of upcoming state transition. They extracted the coefficient of variation of noise-induced fluctuations of model variables while forwarding the system towards the SN point by gradually increasing the predator mortality rate *μ*_*P*_ as a control parameter. Their results showed that only *J* population showed growth in fluctuation amplitude. Since they did not observe any fluctuation growth for *P* and *A* populations they concluded that a catastrophic collapse can occur quietly, and that the claims for universality of early signs of upcoming state transitions are not correct. In the current paper we have investigated the same model prior to SN bifurcation and have elucidated what was missed in the previous analysis. Our results emphasis the importance of performing a full eigenvalue analysis in capturing complete dynamics of the system.

We showed that steady state diagrams of *J* and *P* populations versus *μ*_*P*_ follow an S-bend shape with multi-root regions, while the *A* steady state is a simple linear function of *μ*_*P*_. Using both numerical and theoretical approaches we were able to demonstrate noise-induced early warning signs on both *J* and *P* fluctuations. We identified some guiding principles for reliable detection of critical fluctuations. These include:

- monitoring fluctuation variance instead of coefficient of variation (standard deviation over mean)
- extending numerical experiments to close vicinity of SN point
- reducing noise amplitude when close to SN to reduce risk of state transition

We argued that the linear shape of the *A* steady state diagram cannot support a SN bifurcation, so critical fluctuations in *A* are not expected. This demonstrates that the occurrence of a bifurcation in a high-dimensional system does not necessarily mean that all system variables also undergo the bifurcation. As the first step in tracking early warning signs, one should first identify those model variables undergoing the bifurcation.

We observed that variance of *P* initially decreases, and only increases in close proximity to the SN point. This prompted the question of why variances of *P* and *J* populations behave differently although both have a similar distribution of multi-root steady states supporting SN bifurcation. Boerjlist et al. attempted to answer this question by looking at the direction of dominant eigenvector of the linearized model at SN point [1]. They demonstrated that near the bifurcation, the dominant eigenvector almost exclusively points in the direction of juvenile population axis. They concluded that slowing down only occurs in the direction of the dominant eigenvector. We further analysed the model by looking at the contribution of each variable in dominant eigenvector on wide range of predator mortality rate of 0.4 ≤ *μ*_*P*_ ≤ *μ*_*B*_2__. Our results also confirmed that the dominant eigenvector is largely composed of *J* component near bifurcation point. We also found that the contribution of *J* component increases regularly towards bifurcation point, while the contribution of other two variables mainly has decreasing behaviour.

We extended the work using observability analysis by computing the observability coefficient of the three system variables, revealing that the internal dynamics of the model is best observed from the *J* variable only. System dynamics is only marginally observable using fluctuations in *P* near bifurcation, and almost non-observable via *A* fluctuations. These findings are concordant with the variances of noise-induced fluctuations as early warning signs of upcoming SN bifurcation.

The observability coefficient identifies those system variables which are most representative of whole systems dynamics. Using the observability coefficients to quantify degree of observability—on a 0 to 1 scale—gives a quantitative advantage over the simple eigenvector-based analysis. Observability analysis provides a useful tool in looking for signs of critical fluctuations in complex multivariable models by highlighting those system variables that are sensitive to early signs of catastrophic regime shift. Having established the usefulness of observability analysis in looking for signs of critical fluctuations, there are techniques that would help infer observability from (roughly stationary) experimental data without requiring knowledge of the governing model equations of the underlying system [15].

## Supporting Information

S1 Fig Fluctuation variance prior to saddle-node bifurcation with noise added to *J* population only. (PDF)

S2 Fig Fluctuation variance prior to saddle-node bifurcation with noise added to *P* population only. (PDF)

S1 Appendix Observability. (PDF)

S2 Appendix Observability matrix made from coordinate transformation. (PDF)
