# Supplementary material for: Which System Variables Carry Robust Early Signs of Upcoming Phase Transition? An Ecological Example
Source: PLoS One. 2016 Sep 15;11(9):e0163003. doi: 10.1371/journal.pone.0163003 (PMC5025176; doi:10.1371/journal.pone.0163003)
Supplement: S1 Fig — (PDF) [file pone.0163003.s001.pdf]

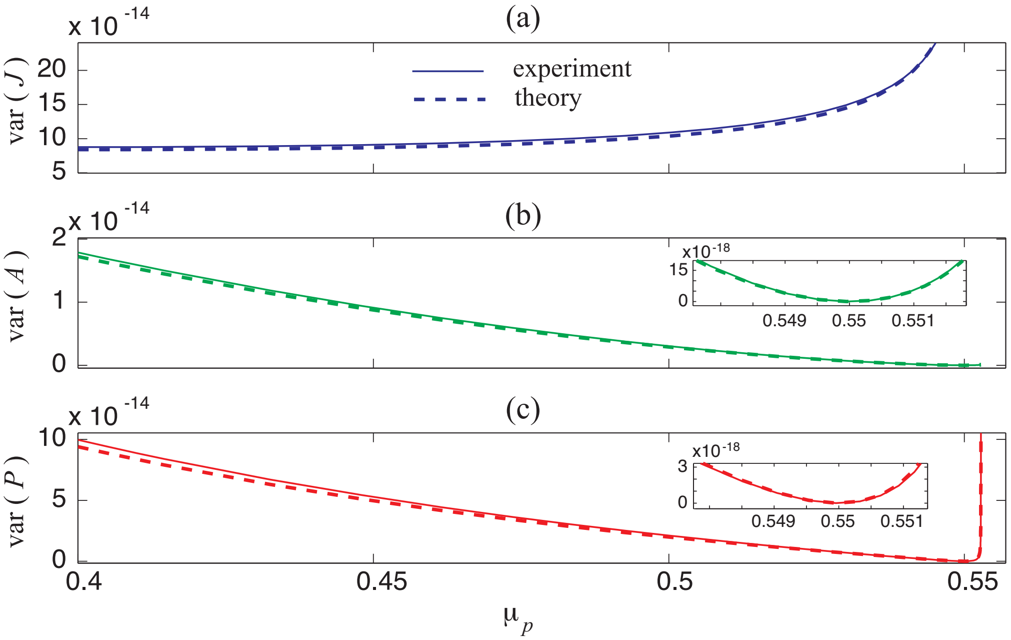

S1 Fig. Fluctuation variance prior to saddle-node bifurcation with noise added to J population only. (a-c) Experimental and theoretical variances are plotted for all three populations while approaching catastrophe. A fixed step Euler method with $\Delta t = 0.01$ time units is used for numerical simulations each for 6000 time units. Starting with $\mu_P = 0.4$, it is incremented in a geometrical way towards the saddle-node point at μ_{B_2}. White noise is added to J population with standard deviation of $\sigma_{\text{noise}} = 2 \times 10^{-6}$.
