# Supplementary material for: Which System Variables Carry Robust Early Signs of Upcoming Phase Transition? An Ecological Example
Source: PLoS One. 2016 Sep 15;11(9):e0163003. doi: 10.1371/journal.pone.0163003 (PMC5025176; doi:10.1371/journal.pone.0163003)
Supplement: S2 Fig — (PDF) [file pone.0163003.s002.pdf]

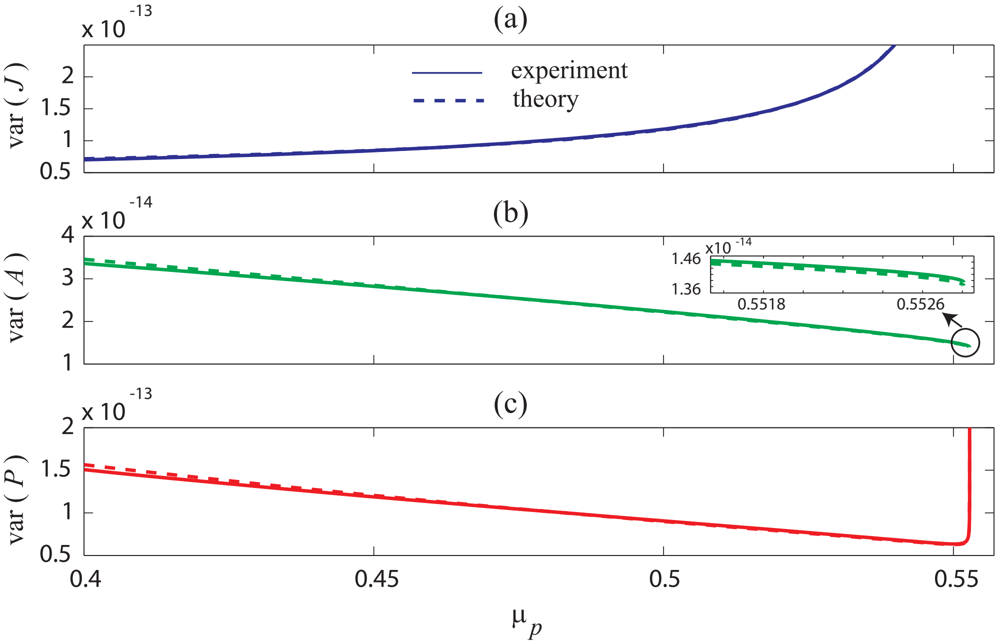

S2 Fig. Fluctuation variance prior to saddle-node bifurcation with noise added to P population only. (a-c) Experimental and theoretical variances are plotted for all three populations while approaching catastrophe.
