# Supplementary material for: Which System Variables Carry Robust Early Signs of Upcoming Phase Transition? An Ecological Example
Source: PLoS One. 2016 Sep 15;11(9):e0163003. doi: 10.1371/journal.pone.0163003 (PMC5025176; doi:10.1371/journal.pone.0163003)
Supplement: S1 Appendix — (PDF) [file pone.0163003.s003.pdf]

S1 Appendix. **Observability.** Consider an m-dimensional system described as $\dot{x}(t) = f(x(t))$, where t is time, $x \in \mathrm{R}^m$ is the vector of state variables, and f is the nonlinear vector field. The observability matrix of the system observed from a scalar time series s is defined as

$$\mathcal{O}_s = \begin{bmatrix} C \\ C\tilde{\mathbf{J}} \\ C\tilde{\mathbf{J}}^2 \\ \vdots \\ C\tilde{\mathbf{J}}^{m-1} \end{bmatrix} \tag{1}$$

where $\tilde{\mathbf{J}}$ is the Jacobian matrix of the system, $s = Cx$ is the observed scalar time series, C is the measurement vector and

$$\tilde{\mathbf{J}}^{n+1} = \left[\frac{\partial \mathcal{L}^n_{f_i} f_i}{\partial x} \right], \qquad i = 1, 2, \ldots, m \tag{2}$$

for $n = 0, \ldots, m-2$, where

$$\mathcal{L}_f f_i(x) = \frac{\partial f_i(x)}{\partial x} f(x) = \sum_{k=1}^{m} \frac{\partial f_i(x)}{\partial x} f_k \tag{3}$$

is the Lie derivative of the ith component of the vector field f. The higher order derivatives can be recursively determined as

$$\mathcal{L}^n_f f_i(x) = \mathcal{L}_f \left[\mathcal{L}^{n-1}_f f_i(x) \right]. \tag{4}$$

The system is observable at point $(s, \dot{s}, \ldots, s^{(m-1)})$ if $\mathcal{O}_s$ is full rank (rank equal to m), that is, if $\mathcal{O}_s^T \mathcal{O}_s$ is nonsingular.
