# Supplementary material for: Which System Variables Carry Robust Early Signs of Upcoming Phase Transition? An Ecological Example
Source: PLoS One. 2016 Sep 15;11(9):e0163003. doi: 10.1371/journal.pone.0163003 (PMC5025176; doi:10.1371/journal.pone.0163003)
Supplement: S2 Appendix — (PDF) [file pone.0163003.s004.pdf]

S2 Appendix. Observability matrix made from coordinate transformation. An equivalent phase portrait of the original system can be reconstructed using any individual variable of the system and its derivatives. Three mappings can be constructed, each corresponding to one of the system variables $J,\ A$ and P,

$$\Phi_J = \begin{cases} X = J \\ Y = \dot{J} = f_1(J, A, P) \\ Z = \ddot{J} = \dfrac{\partial f_1}{\partial J} f_1(J, A, P) + \dfrac{\partial f_1}{\partial A} f_2(J, A, P) + \dfrac{\partial f_1}{\partial P} f_3(J, A, P) \end{cases}$$

$$\Phi_A = \begin{cases} X = A \\ Y = \dot{A} = f_2(J, A, P) \\ Z = \ddot{A} = \dfrac{\partial f_2}{\partial J} f_1(J, A, P) + \dfrac{\partial f_2}{\partial A} f_2(J, A, P) + \dfrac{\partial f_2}{\partial P} f_3(J, A, P) \end{cases} \tag{1}$$

$$\Phi_P = \begin{cases} X = P \\ Y = \dot{P} = f_3(J, A, P) \\ Z = \ddot{P} = \dfrac{\partial f_3}{\partial J} f_1(J, A, P) + \dfrac{\partial f_3}{\partial A} f_2(J, A, P) + \dfrac{\partial f_3}{\partial P} f_3(J, A, P) \end{cases}$$

The Jacobian matrix of the coordinate transformation map Φ_x can be interpreted as observability matrix $\mathcal{O}_x$ where $x \in \{J,\ A,\ P\}$. It should be pointed out that if the Jacobian matrix of Φ_x is singular, then there is no global diffeomorphism between the original state space and the reconstructed one. Hence, a rank-deficient observability matrix says that there are singularities or "blind spots" in the reconstructed space from which we cannot figure out what is going on in the original space just by measuring that particular variable.

The expressions for coordinate transformations of Eqns (1) are

$$
\Phi_J = \begin{cases} X = J \\ Y = \dot{J} = bA - \dfrac{J}{1+J^2} - \mu_J J \\ Z = \ddot{J} = \left[\dfrac{-(1-J^2)}{(1+J^2)^2} - \mu_J\right](bA - \dfrac{J}{1+J^2} - \mu_J J) + b(\dfrac{J}{1+J^2} - AP - \mu_A A) \end{cases}
$$

$$
\Phi_A = \begin{cases} X = A \\ Y = \dot{A} = \dfrac{J}{1+J^2} - AP - \mu_A A \\ Z = \ddot{A} = \dfrac{1-J^2}{(1+J^2)^2}\left[bA - \dfrac{J}{1+J^2} - \mu_J J\right] - (\mu_A + P)\left[\dfrac{J}{1+J^2} - AP - \mu_A A\right] \\ \qquad\qquad -A(cAP - \mu_P P) \end{cases}
$$

$$
\Phi_P = \begin{cases} X = P \\ Y = \dot{P} = cAP - \mu_P P \\ Z = \ddot{P} = cp\left[\dfrac{J}{1+J^2} - A(P + \mu_A)\right] + P(cA - \mu_P)^2 \end{cases} \tag{2}
$$
